# DNA Microarray Platform for Detection and Surveillance of Viruses Transmitted by Small Mammals and Arthropods

**DOI:** 10.1371/journal.pntd.0005017

**Published:** 2016-09-21

**Authors:** Mohd Jaseem Khan, Amanda Cristina Trabuco, Helda Liz Alfonso, Mario Luis Figueiredo, Weber Cheli Batista, Soraya Jabur Badra, Luiz Tadeu Figueiredo, Marco Aurélio Lavrador, Victor Hugo Aquino

**Affiliations:** 1 Laboratory of Virology, Department of Clinical Analyses, Toxicology and Food Sciences, Faculty of Pharmaceutical Sciences of Ribeirao Preto, University of Sao Paulo, Ribeirao Preto, Sao Paulo, Brazil; 2 Laboratory of Virology, Research Center of Tropical Medicine, Porto Velho, Rondonia, Brazil; 3 Center for Virology Research, School of Medicine of Ribeirao Preto, University of Sao Paulo, Ribeirao Preto, Sao Paulo, Brazil; 4 Laboratory of Bioinformatics and Biostatistics, Department of Physics and Chemistry, Faculty of Pharmaceutical Sciences of Ribeirao Preto, University of Sao Paulo, Ribeirao Preto, Sao Paulo, Brazil; University of Texas Medical Branch, UNITED STATES

## Abstract

Viruses transmitted by small mammals and arthropods serve as global threats to humans. Most emergent and re-emergent viral agents are transmitted by these groups; therefore, the development of high-throughput screening methods for the detection and surveillance of such viruses is of great interest. In this study, we describe a DNA microarray platform that can be used for screening all viruses transmitted by small mammals and arthropods (SMAvirusChip) with nucleotide sequences that have been deposited in the GenBank. SMAvirusChip was designed with more than 15,000 oligonucleotide probes (60-mers), including viral and control probes. Two SMAvirusChip versions were designed: SMAvirusChip v1 contains 4209 viral probes for the detection of 409 viruses, while SMAvirusChip v2 contains 4943 probes for the detection of 416 viruses. SMAvirusChip was evaluated with 20 laboratory reference-strain viruses. These viruses could be specifically detected when alone in a sample or when artificially mixed within a single sample. The sensitivity of SMAvirusChip was evaluated using 10-fold serial dilutions of dengue virus (DENV). The results showed a detection limit as low as 2.6E3 RNA copies/mL. Additionally, the sensitivity was one log_10_ lower (2.6E2 RNA copies/mL) than quantitative real-time RT-PCR and sufficient to detect viral genomes in clinical samples. The detection of DENV in serum samples of DENV-infected patients (n = 6) and in a whole blood sample spiked with DENV confirmed the applicability of SMAvirusChip for the detection of viruses in clinical samples. In addition, in a pool of mosquito samples spiked with DENV, the virus was also detectable. SMAvirusChip was able to specifically detect viruses in cell cultures, serum samples, total blood samples and a pool of mosquitoes, confirming that cellular RNA/DNA did not interfere with the assay. Therefore, SMAvirusChip may represent an innovative surveillance method for the rapid identification of viruses transmitted by small mammals and arthropods.

## Author Summary

Viruses transmitted by small mammals and blood-feeding insects represent global threats to humans. Most emergent viral agents are transmitted by these groups; therefore, the development of methods for the detection and surveillance of such viruses is of great interest. Here, we describe a DNA microchip platform (SMAvirusChip) that can be used for screening all known viruses that are transmitted by small mammals and blood-feeding insects. SMAvirusChip was evaluated using 20 laboratory reference-strain viruses, which were specifically detected when present alone in a sample or when artificially mixed within a single sample. SMAvirusChip had a detection limit as low as 2.6E3 RNA copies/mL, which was sufficient to detect viral genomes in clinical samples. The detection of DENV in serum samples of DENV-infected patients (n = 6) and in a whole blood sample artificially contaminated with DENV confirmed the applicability of SMAvirusChip for the detection of viruses in clinical samples. In addition, in a sample composed of a pool of mosquitoes that was spiked with DENV, virus was also detectable. Therefore, SMAvirusChip may serve as an innovative surveillance method for the rapid identification of viruses transmitted by small mammals and blood-feeding insects.

## Introduction

Human activity is responsible for global environmental and climate changes, which can negatively impact human health. Uncontrolled urbanization, deforestation, large-scale agriculture, road construction, dam building, and rapid expansion of global trade and air travel are important factors that have been associated with the spread of viruses, including those transmitted by small mammals and arthropods, which are associated with significantly increased morbidity and mortality rates [1–3]. Viruses transmitted by small mammals belong to the families *Arenaviridae* (*Mammarenavirus* and *Reptarenavirus* genera) and *Bunyaviridae* (*Hantavirus* genus) [2, 4]. The small mammal hosts of these viruses are typically chronically infected; however, the viruses do not appear to cause obvious illness in them. Transmission to humans occurs mainly by inhalation of air contaminated with virus particles shed by infected small mammals in their urine, feces, and saliva. Most infections caused by arenaviruses never go beyond causing a “flu-like” illness, but sometimes these symptoms herald the onset of neurologic diseases (e.g., *Lymphocytic choriomeningitis virus*) or hemorrhagic fevers (e.g., *Junin virus*, *Machupo virus*, *Lassa virus*, *Guanarito virus*, *Sabia virus* and *Lujo virus*) of varying severity [5]. Infection with hantaviruses can progress to hantavirus pulmonary syndrome (e.g., *Sin nombre virus*, *Andes virus*, and *Laguna Negra virus*) in the Americas and hemorrhagic fever with renal syndrome (e.g., *Hantaan virus* and *Dobrava virus*) in Asia and Europe [6]. Arthropod-borne viruses (arboviruses) include the most important emergent and re-emergent viral agents worldwide. The arboviruses belong to seven taxonomic families: *Bunyaviridae* (*Orthobunyavirus*, *Nairovirus* and *Phlebovirus* genera), *Flaviviridae* (*Flavivirus* genus), *Togaviridae* (*Alphavirus* genus), *Reoviridae* (*Orbivirus*, *Seadornavirus* and *Coltivirus* genera), *Rhabdoviridae* (*Vesiculovirus* and *Ephemerovirus* genera), *Orthomyxoviridae* (*Thogotovirus* genus) and *Asfarviridae* (*Asfarvirus* genus). They cause a wide range of infections in humans and domestic and wild animals [7, 8]. More than 150 arboviruses are known to infect humans, and infection most commonly leads to fever, headache and malaise, but encephalitis and hemorrhagic fever may also occur [9–12]. All viruses transmitted by small mammals and arthropods have RNA genomes, with the exception of asfarvirus, which has a DNA genome.

No vaccines or specific antiviral treatments are available for viruses transmitted by small mammals and arthropods, with a few exceptions [e.g., *Yellow fever virus* (YFV), Japanese encephalitis virus (JEV), *Tick-borne encephalitis virus* (TBE) and Junin virus (JUNV)], [12–15]. Therefore, early diagnosis of infection with one of these viruses is of great importance for proper patient management and the rapid implementation of epidemic containment strategies. However, currently available methods for the diagnosis of virus infections are time consuming and expensive, especially when several assays are necessary to identify a virus because only one or a few viruses can be screened per assay using conventional methods. Traditionally, virus isolation has been considered the gold standard for virus diagnosis, but the detection of viral genomic nucleic acids by polymerase chain reaction (PCR) has emerged as an alternative to virus isolation due to its simplicity, rapidity and sensibility. However, PCR does not allow for the simultaneous screening of several virus. High-throughput nucleic acid sequencing methods provide the most in-depth and unbiased information for virus surveillance, but they are very expensive and too time consuming to be used for the routine diagnosis of virus infection. An alternative method for virus surveillance is DNA microarray technology, which enables simultaneous screening of a significantly higher number of viruses than PCR methods and is more economical and faster than high-throughput nucleic acid sequencing methods. Several DNA microarray platforms have been described in the literature for virus detection [16–18]. Therefore, DNA microarray technology could be an alternative for the rapid identification of viruses, especially when conventional virological methods fail to identify a virus.

We describe in this study a DNA microarray platform that could be used for the detection and surveillance of viruses transmitted by small mammals and arthropods.

## Material and Methods

### Database of nucleic acid sequences

The complete and partial nucleotide sequences of all viruses transmitted by small mammals and arthropods that can be found in GenBank were retrieved to design a DNA microarray platform (SMAvirusChip). Sequences deposited in GenBank until September 2013 were used to design the first version of the platform (SMAvirusChip v1) and those deposited until November 2013 were used for the second version (SMAvirusChip v2). The viral species transmitted by small mammals were selected based on the virus taxonomy list of the International Committee on Taxonomy of Viruses (ICTV 2011) [19]. These viruses belong to the *Bunyaviridae* family, *Orthobunyavirus*, *Nairovirus*, *Phlebovirus* and *Hantavirus* genera and the *Arenaviridae* family, *Arenavirus* genus. The virus species transmitted by arthropods were selected from the virus taxonomy list of the ICTV and the Arbovirus Catalog of the Centers for Disease Control and Prevention (CDC) (https://wwwn.cdc.gov/Arbocat/Default.aspx). These viruses belong to the *Flaviviridae* family, *Flavivirus* genus; *Reoviridae* family, *Orbivirus*, *Coltivirus* and *Seadornavirus* genera; *Rhabdoviridae* family, *Vesiculovirus* and *Ephemerovirus* genera; *Togaviridae* family, *Alphaviru*s genus; *Orthomyxoviridae* family, *Thogotovirus* genus; and *Asfarviridae* family, *Asfarvirus* genus.

### Oligonucleotide probes and microarray design

To design specific probes for virus species identification, open reading frame sequences of several representative isolates from each virus species were aligned using CLC Main Workbench (CLC bio, USA) software. The alignments were used to design 60-mer candidate probes using the CLC Main Workbench or SCPrimer software [20], which selected the probes from conserved regions. Candidate probes with a melting temperature (T_m_) of 60–80°C and a G/C content of 40–60% were analyzed using Basic Local Alignment Search Tool (BLAST) software (NCBI, http://blast.ncbi.nlm.nih.gov/Blast.cgi) to perform a similarity search. Probes showing ≥95% identity to a corresponding virus species and <80% identity to non-target sequences were selected. The SMAvirusChip was constructed with 60-mer oligonucleotide probes, which were synthesized on a 75 mm x 25 mm glass slide by applying an inkjet deposition system in a format composed of eight identical sub-arrays, each having 15,000 probes (Agilent Technologies, Palo Alto, CA). All hybridizations involved fluorescently labeled synthetic oligonucleotides that were complementary to positive control probes, which were replicated in 200 spots scattered in different zones of each sub-array. In addition, synthetic oligonucleotides that do not hybridize to any other sequence found in nature were replicated in 78 spots scattered in different zones of each sub-array to serve as negative control probes. Nucleotide sequences of positive and negative control probes are not shown in this study because they were designed by the manufacturer (Agilent) and are company confidential information.

### Viruses

Laboratory viruses were propagated in C6/36 or Vero cells using Leibovitz's L-15 medium containing 10% fetal bovine serum (FBS) (Cultilab, Brazil) while following biocontainment regulations and guidelines. In addition, a Zika virus isolate from a febrile patient was obtained from the brain of an infected baby mouse (Table 1). All the viruses were obtained from the Virology Collection at the Virology Research Center of the Medical School of Ribeirao Preto, University of Sao Paulo.

**Table 1:** Laboratory viruses used in this study.

Family	Genus	Virus	Abbreviation	Strain	Maintain in (cell line)
*Bunyaviridae*	*Orthobunyavirus*	*Guaroa virus*	GROV	BeAn 277	C6/36
		*Oropouche virus*	OROV	BeAn19991	C6/36
	*Hantavirus*	*Rio Mamore virus*	RIOMV	OM 556	VERO E6
*Flaviviridae*	*Flavivirus*	*Bussuquara virus*	BSQV	BeAn 4073	C6/36
		*Cacipacore virus*	CPCV	BeAn 327600	C6/36
		*Dengue virus* type 1	DENV-1	Hawaii	C6/36
		*Dengue virus* type 2	DENV-2	New Guinea C	C6/36
		*Dengue virus* type 3	DENV-3	H87	C6/36
		*Dengue virus* type 4	DENV-4	H 241	C6/36
		*Iguape virus*	IGUV	Span 71 686	C6/36
		*Ilheus virus*	ILHV	BeH 7445	C6/36
		*Rocio virus*	ROCV	SPH 34675	C6/36
		*St*. *Louis encephalitis virus*	SLEV	BeH 356964	C6/36
		*West Nile virus*	WNV	NY99-4132	C6/36
		*Yellow fever virus*	YFV	17D (vaccine)	C6/36
		*Zika virus*	ZIKAV	*	Baby mouse brain
*Rhabdoviridae*	*Vesiculovirus*	*Piry virus*	PIRYV	BeAn24232	C6/36
*Togaviridae*	*Alphavirus*	*Chikungunya virus*	CHIKV	ATCC VR 64	C6/36
		*Mayaro virus*	MAYV	BeAn243	C6/36
		*Mucambu virus*	MUCV	ATCC VR 580	C6/36

*Isolated from patient.

### Clinical samples

DENV-negative samples (n = 28) and DENV-positive samples (n = 6; DENV-1 = 2, DENV-2 = 1, DENV-3 = 1 and DENV-4 = 2) were obtained from the biorepository of the Laboratory of Virology of the Faculty of Pharmaceutical Sciences of Ribeirao Preto, University of Sao Paulo (Biorepository approval number: CEP/FCFRP n° 006/2013). The presence of DENV genomes in these clinical samples was confirmed by real-time RT-PCR as previously described [21]. In addition, serum (n = 6) and whole blood (n = 9) samples of suspected cases of malaria (n = 9) that were negative for malaria based on capillary blood tests (Giemsa stain test) were also included in the study. Clinical samples from malaria-suspected patients (MSP) were collected at the Center of Research in Tropical Medicine, Porto Velho, Rondonia. Written informed consent was obtained from patients or guardians of children from Porto Velho who participated in this study. The whole blood samples were frozen at -80°C overnight for cell lysis and then clarified by centrifugation in a microcentrifuge at 4°C and 12,000 rpm for five minutes. The clarified blood and serum samples were stored at -80°C until use. This study was approved by the Ethical Committee of the Faculty of Pharmaceutical Sciences of Ribeiao Preto, University of Sao Paulo (CEP/FCFRP n° 313/2013).

### Mosquito samples

Unengorged female mosquitoes (*Culicidae*, Diptera) captured in the urban area of Monte Negro County, Rondonia State, North Region of Brazil, were kindly donated by Dr. Edison Luiz Durigon. Mosquitoes were identified based on morphologic characteristics after being placed in a CO_2_ atmosphere [22]. Female *Aedes aegypti* mosquitoes (n = 10) that were captured in the same place on the same day were pooled. The mosquito pool samples (MPS) (n = 4) were triturated in a 1.5 ml microcentrifuge tube containing glass beads and 1.5 ml of 4% bovine plasma albumin (BPA) in phosphate-buffered saline by vortexing the tube for 1–2 minutes. The mosquito homogenate was clarified by centrifugation in a microcentrifuge at 4°C and 12,000 rpm for five minutes. The clarified mosquito homogenate was transferred to a clean microcentrifuge tube and stored at -80°C until used.

### Microarray experiments

#### SMAvirusChip v1

cDNA of the purified RNA was synthesized using random primers with eight nucleotides linked to a specific artificial sequence (5’- GTT TCC CAG TAG GTC TCN NNN NNN N-3') [18, 23]. The reaction mixture for cDNA synthesis contained 12 μl of RNA, 1.2 mM of the random primers (Invitrogen, USA), 2 μL of a 1:5000 dilution of the positive RNA control (Agilent, USA), 0.4 mM dNTPs (Invitrogen, USA), 40 U RNAse inhibitor (RNAseOUT, Invitrogen, U.S.), 4 mM DTT, 200 U of M-MLV reverse transcriptase (Invitrogen, USA) and 5 μl of 5X buffer (250 mM Tris-HCl [pH 8.3], 375 mM KCl, 15 mM MgCl_2_) in a total volume of 25 μl. The mixture was incubated at 25°C for 10 minutes, followed by incubation at 37°C for 2 hours and, finally, incubation at 85°C for 5 minutes. The cDNA was amplified by PCR using a 1:9 mixture of the random primers and a primer targeting a specific primer sequence on the random primer (5’-CGC CGT TTC CCA GTA GGT CTC-3') [16, 18]. In addition to the primers, the PCR reaction mixture contained 4 μl of cDNA, 2 mM MgCl_2_, 0.2 mM dNTP, 5 U of Platinum Taq DNA Polymerase (Invitrogen, USA), and 5 μl of 10X buffer [SO_4_ 600 mm Tris (pH 8.9), NH_4_ SO_4_ 180 mM] in a total volume of 50 μl. Amplification was performed using a Thermal Cycler MyCycler (Bio-Rad, USA). The reaction mixture was subjected to 8 cycles of 94°C for 30 seconds, 25°C for 1 min and 72°C for 1 minute, followed by 35 cycles at 94°C for 30 seconds, 55°C for 1 minute, and 72°C for 1 minute. PCR products were purified using Wizard SV Gel and a PCR Clean-Up System (Promega, USA) following the manufacturer's recommendations. The PCR products were labeled with a 3DNA Array 900MPX kit (Genisphere, Hatfield, PA, USA) following manufacturer’s recommendations. Briefly, the PCR products were tagged with a capture sequence for 3DNA dendrimers, which contain Cy3 dye as fluorescent reporter molecule. The tagged PCR products were added to hybridization buffer (Genisphere, USA), heated at 80°C for 10 minutes, and then added to each sub-array on the SMAvirusChip for hybridization at 65°C overnight in an Agilent Hybridization oven (Agilent Technology, USA) at a rotation speed of 15 rpm. After hybridization, the slides were washed (2X SSC, 0.2% SDS) three times to remove non-hybridized PCR products. The 3DNA dendrimers were mixed with the hybridization buffer (Genisphere, USA) and then added to the SMAvirusChip for hybridization at 65°C for 4 hours at a rotation speed of 15 rpm followed by a washing step as described above.

#### SMAvirusChip v2

Target cRNA synthesis and hybridization were performed using Agilent’s Low Input Quick Amp WT Labeling kit, one-color (Agilent Technology, USA), according to the manufacturer’s instructions. Briefly, total RNA purified from the samples was incubated at 65°C for 10 min with a WT primer mix and a 1:5000 dilution of a positive RNA control (Agilent, USA). cDNA master mix (5X first-strand buffer, 0.1 M DTT, 10 mM dNTP mix, RNase-Out, and MMLV-RT) was added to the mixture, which was then incubated at 40°C for 2 hours. Following this, reverse transcription and dsDNA synthesis were terminated by incubating the mixture at 70°C for 15 min. Transcription master mix was prepared per the manufacturer’s protocol (4X Transcription buffer, 0.1 M DTT, NTP mix, 50% PEG, RNase-Out, inorganic pyrophosphatase, T7-RNA polymerase, and Cyanine 3/5-CTP). Transcription of dsDNA was performed by adding the transcription master mix to the dsDNA reaction samples and incubating at 40°C for 2 hours. The transcription reaction product was purified using a MEGAclear Kit (Amion, USA) according to the manufacturer’s instructions. Cyanine 3-labeled cRNA was fragmented by adding 10X blocking agent and 25X fragmentation buffer and incubating at 60°C for 30 min. Fragmented cRNA from each sample was resuspended in 2X hybridization buffer and directly pipetted onto each sub-array on the SMAvirusChip v2 microarray. The arrays were hybridized at 65°C with a rotation speed of 10 rpm for 17 h in an Agilent Hybridization oven (Agilent Technology, USA). The SMAvirusChip slides were then washed with Gene Expression Wash Buffer 1 for 1 min and with Gene Expression Wash Buffer 2 for 1 min. The slides were added to a rack containing Stabilization and Drying Solution for 30 seconds.

### Data collection and analysis

The SMAvirusChip slides were scanned using an Axon GenePix 4000B scanner (Molecular Devices, USA) with a 532-nm laser and a 10-μm resolution. The median fluorescence intensity of each spot with local background subtraction (median F532 –median B532) was calculated from the scanned images using GenePix Pro 7 software (Molecular Devices, USA). We developed a SMAvirusChip Analysis Form (RAF) using Microsoft Excel software for data processing and analysis. The RAF contains an algorithm similar to that used by DetectiV software [24]. All the raw data were normalized against the negative control probes, which were randomly distributed in 78 spots on each sub-array. To accomplish this, the fluorescence intensity of each spot/probe was divided by the mean fluorescence intensity of the negative control probes. After normalization, all values <1 were transformed to 1. The normalized data were log_2_-transformed to reduce variability. The spots containing the probes of each virus species were grouped, and the mean signal intensity of all groups was calculated. The hypothesis that the mean signal intensity of the group of probes corresponding to each virus species is equal to the mean signal intensity of the negative control probes was tested using Welch’s t-test, a variant of the t-test. This test is useful when two samples have unequal variances, such as for the data obtained with the SMAvirusChip platform [25]. A virus was considered present in an analyzed sample when the mean signal intensity of the group of probes was significantly (p≤0.05) higher than the mean signal intensity of the negative control probes and showed a normalized mean intensity of ≥1, i.e., it was at least twofold higher than the mean signal intensity of the negative control probes. The data discussed in this manuscript have been deposited in NCBI's Gene Expression Omnibus and are accessible through GEO Series accession numbers GSE81393, GSE81391 and GSE81392 (https://www.ncbi.nlm.nih.gov/geo/browse/?view=series&submitter=46842) [26].

### Sensitivity of the SMAvirusChip

The sensitivity of SMAvirusChip was evaluated using a DENV-1 sample with a titer of 2.6E10 RNA copies/mL. The viral titer was determined with quantitative real-time RT-PCR as previously described [27]. The sensitivity of SMAvirusChip for virus detection was compared to that of a real-time RT-PCR. To determine the sensitivities of both methods, 10-fold serial dilutions of viral RNAs were assessed. The lower limits of detection for SMAvirusChip and real-time RT-PCR were defined as the last dilution where viral RNA was detected, and they were expressed as RNA copies/mL.

### Detection of mixture of viruses with SMAvirusChip

The ability of SMAvirusChip to detect a mixture of viruses was evaluated by analyzing four pools of viruses: 1) viruses from different families, including BSQV (*Flaviviridae*), MAYV (*Togaviridae*) and PIRYV (*Rhabdoviridae*); 2) viruses of the *Flavivirus* genus, including DENV-2, ROCV, and SLEV; 3) the four DENV serotypes; and 4) viruses that have caused epidemics in Brazil, including CHIKV, DENV-1 and ZIKV. Viral RNAs were purified and used at equal volumes to prepare the pools, which were assessed with the microarray assay as described above.

## Results

### Probe design and selection

We retrieved ~9000 viral target sequences from GenBank to design probes for the detection of viruses transmitted by small mammals and arthropods. Complete or partial nucleotide sequences (as many as possible) of the open reading frame for each virus species were aligned and used to design probes from highly conserved regions, which were selected by either the CLC Main Workbench or SCPrimer software. Probes showing ≥95% identity to the corresponding virus species and <80% identity to non-target sequences were selected. Up to 10 probes were selected for viruses presenting a single genomic segment, and up to three probes per segment were selected for those presenting more than one genomic segment. For *Reoviridae* family viruses, which have 10–12 RNA genomic segments, probes were designed for the genomic segments encoding the structural proteins VP2 and VP5 and the non-structural protein NS3. Fewer probes were selected for viruses with few sequences available in GenBank. Two versions of the SMAvirusChip were designed: SMAvirusChip v1 contains 4209 probes for 409 virus species (109 viruses transmitted by small mammals and 300 arboviruses), and SMAvirusChip v2 contains 4943 probes for 416 virus species (112 viruses transmitted by small mammals and 304 arboviruses) (S1 Table). A DNA microarray slide with eight identical sub-arrays containing viral probes that were replicated at least three times to complete the array with 15,000 probes, including positive and negative control probes, was designed. The number of probes selected for each virus species multiplied by the number of replicates represents the total number of probes used for each virus species in each sub-array. This number was also used for statistical analysis.

### Data processing and analysis

**Fig 1:**
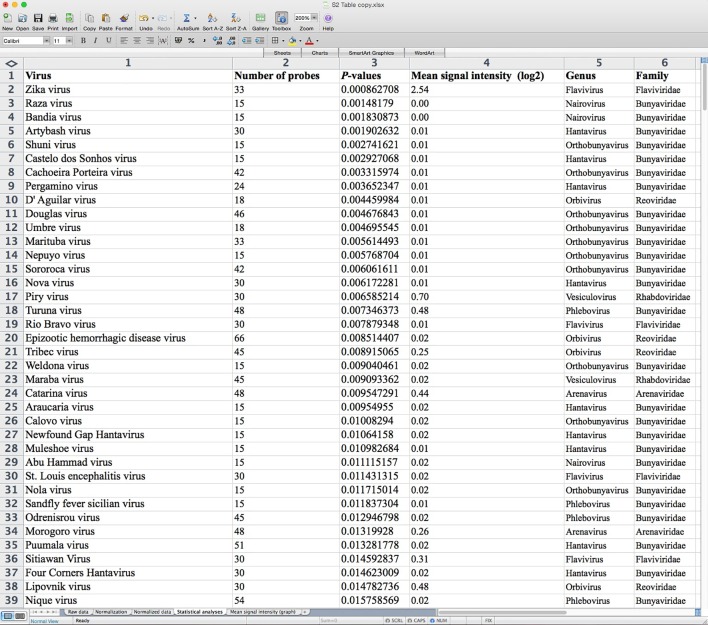
SMAvirusChip v2 form (RAF) showing the results of statistical analysis of an array hybridized with ZIKV.

Microarray data were processed and analyzed using RAF, a user-friendly analysis algorithm prepared with Microsoft Excel software. The raw data corresponding to each probe’s signal intensity were copied and pasted in the matching column in RAF and sorted alphabetically. Then, the normalized data, the mean probe intensity (table and graph) and the results from statistical analysis could be displayed and easily visualized in several spreadsheets within RAF. Fig 1 shows an example RAF spreadsheet displaying the results of statistical analysis of an array hybridized with ZIKV. The viruses were sorted in ascending order according to *P*-value. Only the probes for ZIKV presented a mean signal intensity higher than 1 (mean signal intensity = 2.54), which was significantly different (*P*-value = 8.62708E-9) from the mean signal intensity of the negative control probes.

The entire RAF document for the array hybridized with ZIKV can be seen in S2 Table.

### Identification of laboratory reference viruses

We initially tested SMAvirusChip with 20 laboratory reference-strain viruses obtained from C6/36 and Vero E6 cell cultures and from the brain of a baby mouse (Table 2). In addition, RNAs extracted from uninfected C6/36 or Vero E6 cell cultures were used as controls to analyze cross-hybridization. RNA from each viral and control sample was hybridized to the SMAvirusChip in separate array experiments. The data obtained in the microarray experiments were analyzed with RAF, which showed that the group of probes for each virus tested in each array was the only group showing a mean of signal intensity ≥1 (a value at least double the mean signal intensity of the negative control probes) and that this value was significantly higher (*P*-value ≤0.05) than the mean signal intensity of the negative control probes. In contrast, no significant difference was observed between the mean signal intensity of the group of probes of any virus species and the mean signal intensity of the negative control probes when RNA extracted from uninfected cells was used. This last result, together with the specific detection of ZIKV in the brain of a baby mouse, confirms that cellular DNA/RNA does not interfere with the microarray assay.

**Table 2:** Results of statistical analyses of reference viruses and control samples tested with the SMAvirusChip platform.

	SMAvirusChip v1			SMAvirusChip v2		
Virus	N° of probes	*P-*values	Mean signal intensity (log2)	N° of probes	*P-*values	Mean signal intensity (log2)
BSQV	36	4.1E-13	3.07	-	-	-
CPCV	30	3.5E-08	4.42	-	-	-
CHIKV	34	1.0E-4	2.15			
DENV-1	36	1.1E-17	4.38	30	9.6E-07	1.44
DENV-2	36	3.1E-09	3.51	30	1.3E-21	6.45
DENV-3	39	1.1E-08	3.23	30	3.7E-24	6.75
DENV-4	31	1.5E-07	2.19	30	1.4E-11	2.97
GROV	36	3.2E-11	2.34	-	-	-
IGUV	39	3.8E-12	3.18	-	-	-
ILHV	38	2.1E-24	4.22	-	-	-
MAYV	37	1.9E-06	1.89	-	-	-
MUCV	8	5.0E-3	2.35	-	-	-
OROV	82	5.2E-24	2.74	-	-	-
PIRYV	40	5.4E-08	1.57	-	-	-
RIOMV	53	2.0E-06	1.27	-	-	-
ROCV	33	1.7E-05	2.31	-	-	-
SLEV	34	8.2E-14	3.63	-	-	-
WNV	34	1.2E-10	3.83	-	-	-
YFV	36	8.1E-12	3.40	-	-	-
ZIKV	-	-	-	33	8.6E-09	2.54
C6/36	-	ND	ND	-	ND	ND
Vero E6	-	ND	ND	-	-	-

ND = not detected

### Detection of mixture of viruses

Co-infection with more than one virus is a natural event that can be seen in individuals during epidemic outbreaks. Therefore, we tested the ability of SMAvirusChip to detect a mixture of viruses within a single sample. In this experiment, we analyzed four pools of viruses that were artificially mixed: 1) viruses from different families, including BSQV (*Flaviviridae*), MAYV (*Togaviridae*) and PIRYV (*Rhabdoviridae*); 2) *Flavivirus* genus viruses, including DENV-2, ROCV, and SLEV; 3) the four DENV serotypes; and 4) viruses that have caused epidemics in Brazil, including CHIKV, DENV-1 and ZIKV. The microarray experiments were performed as described above and analyzed with RAF, which showed that all viruses were specifically detected in each of the four pools (Table 3). The entire RAF document for the array hybridized with DENV-1, CHIKV and ZIKV can be seen in S3 Table as an example.

**Table 3:** Results of statistical analyses of pools of viruses analyzed with SMAvirusChip.

Pool	Virus	Mean signal intensity (log_2_)	*P-*values	SMAvirusChip (version)
	BUSV	1.83	5.2E-07	
1	MAYV	1.0	5.9E-4	1
	PIRV	2.81	3.0E-10	
	DENV- 2	1.54	9.2E-05	
2	ROCV	1.55	1.5E-4	
	SLEV	1.41	1.7E-4	1
	DENV-1	4.16	6.9E-13	
	DENV-2	5.01	2.2E-16	
3	DENV-3	5.09	1.1E-17	1
	DENV-4	3.67	1.6E-08	
	CHIKV	8.47	4.9E-19	
4	DENV-1	2.75	1.3E-06	2
	ZIKV	2.87	2.4E-07	

### Sensitivity of the SMAvirusChip

The lower limit of detection for SMAvirusChip v1 was determined using DENV-1 (2.6E10 RNA copies/mL) collected from the supernatant of an infected C6/36 cell culture. Microarray experiments were performed with 10-fold serial dilutions of purified RNA and compared with quantitative real-time RT-PCR data. SMAvirusChip v1 was able to detect as few as 2.6E3 RNA copies/ml, a sensitivity one log_10_ lower than that measured for quantitative real-time RT-PCR (2.6E2 RNA copies/ml).

### Detection of virus in serum samples

To determine the applicability of SMAvirusChip v1 for the detection of viruses in clinical samples, sera collected from six DENV-infected patients (DENV-1 = 2, DENV-2 = 1, DENV-3 = 1, and DENV-4 = 2) and 28 suspected cases of DENV that tested negative for DENV infection by real-time RT-PCR were used. All DENV serotypes were specifically detected in the sera of infected patients (Table 4), while no viruses were detected in the dengue-suspected cases. The viral loads in the serum samples of the dengue-infected patients were determined by real-time RT-PCR.

**Table 4:** Dengue patient sera analyzed with SMAvirusChip v1.

Patient ID	Infecting virus	Detected virus	Mean signal intensity (log2)	*P-*values	Viral load (RNA copies/mL)
RP_45_2010	DENV-1	DENV-1	3.10	3.6E-08	6.3E08
RP_52_2010	DENV-1	DENV-1	1.19	3.9E-05	2.0E06
RP_14_2010	DENV-2	DENV-2	1.49	1.0E-04	2.0E08
RP_15_2010	DENV_3	DENV_3	6.40	7.1E-21	6.3E06
RP_2_2013	DENV_4	DENV_4	1.46	3.0E-04	6.9E05
RP_3_2013	DENV_4	DENV_4	1.66	7.8E-05	1.1E06

### Screening of serum and total blood samples from malaria-negative samples and pool of mosquitoes

We used SMAvirusChip v1 to screen for the presence of viruses transmitted by small mammals and arthropods in patients suspected to have malaria who tested negative for malaria by capillary blood test. We also used this assay to screen for the presence of viruses in mosquitoes. The microarray experiments were performed using serum (n = 6) and whole blood (n = 9) samples from the malaria-suspected patients (n = 9) and pools of mosquitoes (n = 4). All clinical and mosquito samples were negative for virus genomes detection. We also spiked one whole blood sample and one pool of mosquitoes with DENV-2 to evaluate the ability of SMAvirusChip to detect viruses in these sample types. Total RNA purified from these samples was analyzed with SMAvirusChip v1, which showed the specific detection of DENV-2 (Table 5).

**Table 5:** Detection of DENV-2 spiked in a whole blood sample and a pool of mosquitoes with SMAvirusChip v1.

Sample	Samples ID	Mean signal intensity (log2)	*P*- value s
Blood	MSP_PV-15_DENV-2	1.0	3.1E-5
Pool of mosquitoes	PMS-5_DENV-2	1.72	3.0E-6

## Discussion

DNA microarray technology has recently emerged as a high-throughput method for screening numerous pathogens in a single assay. In this study, we designed a highly specific and sensitive DNA microarray platform for the detection and surveillance of viruses transmitted by small mammals and arthropods. Several of the screened viruses represent important threats to human health, while others have the possibility of becoming emergent. DENV (*Flavivirus* genus), which is transmitted mainly by *Aedes aegypti* and *Aedes albopictus*, is the most important arbovirus worldwide, with more than 390 million human infections occurring every year [13]. In addition to the four DENV serotypes, *Chikungunya virus* (*Alphavirus* genus) and *Zika virus* (*Flavivirus* genus) were recently introduced in the Americas and have become important causes of morbidity and mortality in the region [28]. Zika virus has been associated with increased incidences of Guillain-Barré syndrome and microcephaly [29–34]. SMAvirusChip was capable of detecting these three viruses, suggesting that this platform can be used for the surveillance of these important arboviruses.

Due to the circulation of several viruses transmitted by arthropods in tropical and sub-tropical countries, there is a risk of co-infection with more than one virus. Several studies have detected humans presenting with co-infection with two DENV subtypes [35–38], DENV and CHIKV [39], or WNV and JEV [40]. National surveillance systems use virological methods capable of detecting only one virus per assay, leading to a proven underestimation of the number of co-infections. In the current study, SMAvirusChip was able to detect several viruses artificially spiked into a single sample, including a mixture of the four DENV subtypes as well as a mixture of DENV, ZIKV and CHIKV, which are transmitted by the same vector (*Aedes aegypti*) and are currently causing epidemics in the Americas, especially in Brazil. This suggests that the SMAvirusChip platform could be used as a surveillance system to detect individuals infected with either a single or multiple viruses.

We searched for viruses transmitted by small mammals and arthropods in sera from dengue-suspected cases, but no viruses were detected. This result is in agreement with a study performed in Nicaragua, which showed that dengue-suspected patients were not infected with arboviruses or viruses transmitted by small mammals. Instead, they were mainly infected with herpesviruses [41]. Although there is a report of other arboviruses circulating during dengue epidemics [42], this event seems to be rare, which might explain why we did not detect any viruses transmitted by small mammals and arthropods in the dengue-suspected cases. We were also unable to detect any viruses in malaria-suspected patients and in mosquitoes, potentially because of the low number of samples tested.

DNA microarray technology has previously been used to detect some groups of arboviruses and viruses transmitted by small mammals, specially viruses pathogenic to humans within the families *Togaviridae*, *Flaviviridae*, *Bunyaviridae* and *Arenaviridae*, [43–48]. However, it is well known that non-pathogenic viruses can easily become emergent viruses; for example, Rio Mamore virus was previously considered non-pathogenic to humans, but recent fatal hantavirus pulmonary syndrome cases associated with this virus have been reported in Peru, French Guiana and Brazil [49–51]. Therefore, pathogenic and non-pathogenic viruses should be included in surveillance systems, which is why we included all viruses transmitted by small mammals and arthropods, regardless of whether they are pathogenic or non-pathogenic to humans, in the SMAvirusChip platform.

SMAvirusChip v1 was highly sensitive (2.6E3 RNA copies/mL), showing a sensitivity only one log_10_ lower than that of a real-time RT-PCR, which has been used to detect DENV in serum, saliva, and urine samples [21, 27, 52, 53]. We recently determined the viral loads in 72 DENV-infected children and found that the average viral load in serum samples was 3.5E3 RNA copies/mL [52]; in addition, in the current study, we detected DENV genomes in six patients, who showed an average viral load of 1.4E8 RNA copies/mL. Taken together, these data suggest that the SMAvirusChip platform is sufficiently sensitive to detect viral genomes in clinical samples. In agreement with our results, other studies have also found that DNA microarray technology is suitable for the detection of viral genomes in clinical samples [18, 54, 55]. Although no viruses were detected with the SMAvirusChip in human total blood samples and pools of mosquitoes, these results show that probes contained in the microarray does not cross-hybridize to human or mosquitoes DNA/RNAs, suggesting these samples could be used for searching virus genomes, which was supported by the detection of DENV-2 spiked in a total blood and in a pool of mosquitoes. However, further experiments with viruses spiked in human total blood and pool of mosquitoes with different concentration of DNA/RNA are needed to confirm that cellular nuclei acids do not interfere with virus detection in the microarray assay.

The high specificity of SMAvirusChip relies on the strategy used for probe design. Probes were selected from highly conserved regions after aligning several genomic sequences from each virus species. Furthermore, long probes (60-mers) were used, which has been shown to achieve greater sensitivity and specificity when detecting target sequences than the use of short probes (25- to 45-mers) [56, 57]. In this study, random primers were used for cDNA synthesis and PCR to permit amplification of any virus present in the clinical samples and to increase the sensitivity of SMAvirusChip v1. Random genomic amplification has been successfully used by other authors when searching for several groups of viruses, as well as for the detection of bacteria, fungi and protozoa [17, 18, 23, 58, 59]. In contrast, other studies have described the use of group-specific primers to amplify the genomes of target viruses prior to microarray assay [43, 60]. Such primers may not detect target sequences with mutations, which occur frequently in RNA viruses due to the lack of proofreading activity in viral RNA polymerases.

During the development of this work, Genisphere stopped the production of their labeling 3DNA Array 900MPX kit, which was used for SMAvirusChip v1. Therefore, we used a different labeling kit for SMAvirusChip v2 (the Low Input Quick Amp WT Labeling kit, one color). This labeling kit uses RNA as a target molecule, which is why we did not include random genomic amplification in SMAvirusChip v2. In the future, we plan to standardize a random PCR amplification method for viral genomes to be used with the Low Input Quick Amp WT Labeling kit, one color, thus maintaining the sensitivity of the test.

Data analysis is an essential component of using a DNA microarray platform for pathogen detection. Several groups have developed analysis algorithms targeting their own platforms that in some cases can be adapted to other platforms [18, 23, 58, 61, 62]. The results obtained with each analysis algorithm depend on the strategy used for probe selection. Most of the platforms described in the literature have used probes selected from highly conserved regions of viruses within a specific genus or family to detect both known and new viruses; therefore, probes often cross-hybridize to multiple related genome sequences, making it difficult to correctly identify single viruses or mixtures of viruses present in clinical samples because the output of the algorithms is usually a single list of taxa ranked by some score or by *P*-values. We aimed to design the SMAvirusChip platform to enable the surveillance of known viruses, which is why we selected highly specific probes (>95% identity) for each virus species. Although some individual probes showed cross-hybridization with non-target viruses, the group of probes specific to the individual viruses or mixtures of viruses that were present in the samples were the only ones showing a mean signal intensity ≥1, which was significantly higher than the mean signal intensity of the negative control probes. This allowed clear identification of the virus/viruses present in the samples. In addition, we developed the SMAvirusChip Analysis Form (RAF) to facilitate data processing and analysis in a user-friendly environment. The RAF uses Microsoft Excel software, which is widely used in informatics. The manual entering of data on the RAF does not require additional devices or software besides a regular personal computer with Microsoft Office and a RAF template in Excel format. The user needs only to copy and paste the raw signal intensities of probes, sorted alphabetically, into the RAF to visualize all the results in several spreadsheets included in the form. The analysis algorithm used in the RAF is similar to that used in DetectiV software [24], which was written in R, a statistical programming language [63]. However, the R package requires users to have some experience in coding in R because it lacks a graphical interface; therefore, DetectiV software is difficult for non-experienced users.

Most emergent viruses transmitted by arthropods and small mammals over the past 50 years were caused by known viruses that were previously detected in their natural hosts or caused sporadic infections in humans. These include *Dengue virus*, *West Nile virus*, *Zika virus*, *Chikungunya virus*, and *Rio Mamore virus*, among others. Therefore, we were interested in the development of a DNA microarray platform that could enable the surveillance of known viruses because we believe that the emergence of viruses transmitted by arthropods and small mammals would more likely be caused by known viruses compared to new ones. We suggest that DNA microarray platforms designed with highly specific probes for known virus species be used as surveillance systems and that only when these platforms fail should next-generation sequencing methods be used to identify new viruses, thus reducing the cost of surveillance. Between August and September of this year, the Olympics and Paralympic games will held in Rio de Janeiro, Brazil, representing a risk for the introduction of new viruses. The SMAvirusChip platform could play an important role in the early detection of viruses to assist national health authorities.

In summary, we developed a highly specific and sensitive DNA microarray platform that could be used for the detection and surveillance of viruses transmitted by arthropods and small mammals. Although real-time PCR is becoming the gold standard method for diagnosis of viral infections, still face problems for high-throughput screening of multiple viruses. To date, the cost of performing real-time PCR, when a high number of viruses needs to be screened in a single sample, is becoming similar to the cost of the DNA microarray. Therefore, DNA microarray could be a cost-effective method for virus surveillance programs that would help in the identification of newly introduced viruses that can then be detected with conventional methods, thus reducing the costs of diagnosis. The probes used in this platform must be updated periodically to include the ever-increasing number of new viral genome sequences being added to GenBank.

## Supporting Information

S1 Checklist STARD checklist. (DOC)

S1 Table List of viruses. All viruses targeted by SMAvirusChip v1 and v2.
(PDF)

S2 Table SMAvirusChip v2 form (RAF). Data processing and analysis of a sub-array hybridized with ZIKV.
(XLSX)

S3 Table SMAvirusChip v2 form (RAF). Data processing and analysis of a sub-array hybridized with DENV-1, CHIKV and ZIKV.
(XLSX)
