# Supplementary material for: DNA Microarray Platform for Detection and Surveillance of Viruses Transmitted by Small Mammals and Arthropods
Source: PLoS Negl Trop Dis. 2016 Sep 21;10(9):e0005017. doi: 10.1371/journal.pntd.0005017 (PMC5031435; doi:10.1371/journal.pntd.0005017)
Supplement: S1 Checklist — (DOC) [file pntd.0005017.s001.doc]

|  | **Section & Topic** | **No** | **Item** | **Reported on page #** |
| --- | --- | --- | --- | --- |
|  |  |  |  |  |
|  | **TITLE OR ABSTRACT** |  |  |  |
|  |  | **1** | DNA Microarray Platform for Detection and Surveillance of Viruses Transmitted by Small Mammals and Arthropods. |  |
|  | **ABSTRACT** |  |  |  |
|  |  | **2** | Viruses transmitted by small mammals and arthropods serve as global threats to humans. Most emergent and re-emergent viral agents are transmitted by these groups; therefore, the development of high-throughput screening methods for the detection and surveillance of such viruses is of great interest. In this study, we describe a DNA microarray platform that can be used for screening all viruses transmitted by small mammals and arthropods (SMAvirusChip) with nucleotide sequences that have been deposited in the GenBank. SMAvirusChip was designed with more than 15,000 oligonucleotide probes (60-mers), including viral and control probes. Two SMAvirusChip versions were designed: SMAvirusChip v1 contains 4209 viral probes for the detection of 409 viruses, while SMAvirusChip v2 contains 4943 probes for the detection of 416 viruses. SMAvirusChip was evaluated with 20 laboratory reference-strain viruses. These viruses could be specifically detected when alone in a sample or when artificially mixed within a single sample. The sensitivity of SMAvirusChip was evaluated using 10-fold serial dilutions of dengue virus (DENV). The results showed a detection limit as low as 2.6E3 RNA copies/mL. Additionally, the sensitivity was one log10 lower (2.6E2 RNA copies/mL) than quantitative real-time RT-PCR and sufficient to detect viral genomes in clinical samples. The detection of DENV in serum samples of DENV-infected patients (n= 6) and in a whole blood sample spiked with DENV confirmed the applicability of SMAvirusChip for the detection of viruses in clinical samples. In addition, in a pool of mosquito samples spiked with DENV, the virus was also detectable. SMAvirusChip was able to specifically detect viruses in cell cultures, serum samples, total blood samples and a pool of mosquitoes, confirming that cellular RNA/DNA did not interfere with the assay. Therefore, SMAvirusChip may represent an innovative surveillance method for the rapid identification of viruses transmitted by small mammals and arthropods. |  |
|  | **INTRODUCTION** |  |  |  |
|  |  | **3** | Human activity is responsible for global environmental and climate changes, which can negatively impact human health. Uncontrolled urbanization, deforestation, large-scale agriculture, road construction, dam building, and rapid expansion of global trade and air travel are important factors that have been associated with the spread of viruses, including those transmitted by small mammals and arthropods, which are associated with significantly increased morbidity and mortality rates [1-3]. Viruses transmitted by small mammals belong to the families Arenaviridae (Mammarenavirus and Reptarenavirus genera) and Bunyaviridae (Hantavirus genus) [2, 4]. The small mammal hosts of these viruses are typically chronically infected; however, the viruses do not appear to cause obvious illness in them. Transmission to humans occurs mainly by inhalation of air contaminated with virus particles shed by infected small mammals in their urine, feces, and saliva. Most infections caused by arenaviruses never go beyond causing a “flu-like” illness, but sometimes these symptoms herald the onset of neurologic diseases (e.g., Lymphocytic choriomeningitis virus) or hemorrhagic fevers (e.g., Junin virus, Machupo virus, Lassa virus, Guanarito virus, Sabia virus and Lujo virus) of varying severity [5]. Infection with hantaviruses can progress to hantavirus pulmonary syndrome (e.g., Sin nombre virus, Andes virus, and Laguna Negra virus) in the Americas and hemorrhagic fever with renal syndrome (e.g., Hantaan virus and Dobrava virus) in Asia and Europe [6]. Arthropod-borne viruses (arboviruses) include the most important emergent and re-emergent viral agents worldwide. The arboviruses belong to seven taxonomic families: Bunyaviridae (Orthobunyavirus, Nairovirus and Phlebovirus genera), Flaviviridae (Flavivirus genus), Togaviridae (Alphavirus genus), Reoviridae (Orbivirus, Seadornavirus and Coltivirus genera), Rhabdoviridae (Vesiculovirus and Ephemerovirus genera), Orthomyxoviridae (Thogotovirus genus) and Asfarviridae (Asfarvirus genus). They cause a wide range of infections in humans and domestic and wild animals [7, 8]. More than 150 arboviruses are known to infect humans, and infection most commonly leads to fever, headache and malaise, but encephalitis and hemorrhagic fever may also occur [9-12]. All viruses transmitted by small mammals and arthropods have RNA genomes, with the exception of asfarvirus, which has a DNA genome.  No vaccines or specific antiviral treatments are available for viruses transmitted by small mammals and arthropods, with a few exceptions [e.g., Yellow fever virus (YFV), Japanese encephalitis virus (JEV), Tick-borne encephalitis virus (TBE) and Junin virus (JUNV)], [12-15]. Therefore, early diagnosis of infection with one of these viruses is of great importance for proper patient management and the rapid implementation of epidemic containment strategies. However, currently available methods for the diagnosis of virus infections are time consuming and expensive, especially when several assays are necessary to identify a virus because only one or a few viruses can be screened per assay using conventional methods. Traditionally, virus isolation has been considered the gold standard for virus diagnosis, but the detection of viral genomic nucleic acids by polymerase chain reaction (PCR) has emerged as an alternative to virus isolation due to its simplicity, rapidity and sensibility. However, PCR does not allow for the simultaneous screening of several virus. High-throughput nucleic acid sequencing methods provide the most in-depth and unbiased information for virus surveillance, but they are very expensive and too time consuming to be used for the routine diagnosis of virus infection. An alternative method for virus surveillance is DNA microarray technology, which enables simultaneous screening of a significantly higher number of viruses than PCR methods and is more economical and faster than high-throughput nucleic acid sequencing methods. Several DNA microarray platforms have been described in the literature for virus detection [16-18]. Therefore, DNA microarray technology could be an alternative for the rapid identification of viruses, especially when conventional virological methods fail to identify a virus.  We describe in this study a DNA microarray platform that could be used for the detection and surveillance of viruses transmitted by small mammals and arthropods. |  |
|  |  | **4** | The objective of this study was to design a DNA microarray platform for surveillance of all viruses transmitted by small mammals and arthropods (SMAvirusChip) with nucleotide sequences that have been deposited in the GenBank. |  |
|  | **METHODS** |  |  |  |
|  | *Study design* | **5** | Retrospective study |  |
|  | *Participants* | **6** | Dengue patients, suspected dengue patients with negative RT-PCR for dengue and suspected malaria patients with negative blood tests for malaria. |  |
|  |  | **7** | Results from previous tests. |  |
|  |  | **8** | Dengue and suspected dengue cases: University of Sao Paulo, Ribeirao Preto, Sao Paulo, Brazil, 2006-2015.  Suspected malaria cases: Research Center of Tropical Medicine, Porto Velho, Rondonia, Brazil, 2014. |  |
|  |  | **9** | Convenience series |  |
|  | *Test methods* | **10a** | DNA microarray slide containing probes for all viruses transmitted by small mammals and arthropods |  |
|  |  | **10b** | Reverse transcription fallow by the polymerase chain reaction (RT-PCR), real-time method, for dengue and suspected dengue cases. Capillary blood test (Giemsa stain test) for suspected malaria cases. |  |
|  |  | **11** | Dengue virus genome was searched in the serum of suspected dengue cases by real-time RT-PCR. Malaria was searched in suspected malaria cases by Giemsa stain test. |  |
|  |  | **12a** | A virus was considered present in an analyzed sample when the mean signal intensity of the group of probes was significantly (p≤0.05) higher than the mean signal intensity of the negative control probes and showed a normalized mean intensity of ≥1, i.e., it was at least twofold higher than the mean signal intensity of the negative control probes. |  |
|  |  | **12b** | Real –time RT-PCR cycle threshold of 10-40. |  |
|  |  | **13a** | Reference standard results were available to the performers/readers of the index test |  |
|  |  | **13b** | No clinical information and index test results were available  to the assessors of the reference standard |  |
|  | *Analysis* | **14** | This was a proof-of-concept study and not a study of diagnostic accuracy. Therefore, we did not calculate the diagnostic accuracy. We have included only four dengue cases. Viruses transmitted by small mammals and arthropods were searched in suspected dengue (n=28) and suspected malaria (n=9) cases were use for screening with the DNA microarray but no virus was detected. |  |
|  |  | **15** | Not determined. |  |
|  |  | **16** | Not determined. |  |
|  |  | **17** | Not determined. |  |
|  |  | **18** | The sample size was determined at convenience and not for a study of diagnostic accuracy. |  |
|  | **RESULTS** |  |  |  |
|  | *Participants* | **19** | DENV-negative samples (n=28) and DENV-positive samples (n=6; DENV-1=2, DENV-2=1, DENV-3=1 and DENV-4=2) were obtained from the biorepository of the Laboratory of Virology of the Faculty of Pharmaceutical Sciences of Ribeirao Preto, University of Sao Paulo (Biorepository approval number: CEP/FCFRP nº 006/2013). In addition, serum (n=6) and whole blood (n=9) samples of suspected cases of malaria (n=9) that were negative for malaria based on capillary blood tests (Giemsa stain test) were also included in the study. |  |
|  |  | **20** | Baseline demographic and clinical characteristics of participants data have been deposited in NCBI's Gene Expression Omnibus and are accessible through GEO Series accession numbers GSE81393, GSE81391 and GSE81392 (https://www.ncbi.nlm.nih.gov/geo/query/acc.cgi?acc=GSE81393) |  |
|  |  | **21a** | Not determined. |  |
|  |  | **21b** | Not determined. |  |
|  |  | **22** | Not determined. |  |
|  | *Test results* | **23** | Not determined. |  |
|  |  | **24** | Not determined. |  |
|  |  | **25** | None |  |
|  | **DISCUSSION** |  |  |  |
|  |  | **26** | The participants were not selected for a study of diagnostic accuracy. |  |
|  |  | **27** | Further studies are needed to analyze the diagnostic accuracy of the DNA microarray platform. |  |
|  | **OTHER INFORMATION** |  |  |  |
|  |  | **28** | Registration number and name of registry: The data discussed in this study have been deposited in NCBI's Gene Expression Omnibus and are accessible through GEO Series accession numbers GSE81393, GSE81391 and GSE81392. |  |
|  |  | **29** | https://www.ncbi.nlm.nih.gov/geo/query/acc.cgi?acc=GSE81393 |  |
|  |  | **30** | This work was supported by the Sao Paulo Research Foundation (FAPESP), Grant no. 2008/50617-6. MJK was also supported by a FAPESP scholarship (Grant no. 2011/02045-6). VHA holds a CNPq - PQ scholarship (Grant no. 310735/2013-0). The funders had no role in study design, data collection and analysis, decision to publish, or preparation of the manuscript. |  |
|  |  |  |  |  |

STARD 2015

### AIM

STARD stands for “Standards for Reporting Diagnostic accuracy studies”. This list of items was developed to contribute to the completeness and transparency of reporting of diagnostic accuracy studies. Authors can use the list to write informative study reports. Editors and peer-reviewers can use it to evaluate whether the information has been included in manuscripts submitted for publication.

### Explanation

A **diagnostic accuracy study** evaluates the ability of one or more medical tests to correctly classify study participants as having a **target condition.** This can be a disease, a disease stage, response or benefit from therapy, or an event or condition in the future. A medical test can be an imaging procedure, a laboratory test, elements from history and physical examination, a combination of these, or any other method for collecting information about the current health status of a patient.

The test whose accuracy is evaluated is called **index test.** A study can evaluate the accuracy of one or more index tests. Evaluating the ability of a medical test to correctly classify patients is typically done by comparing the distribution of the index test results with those of the **reference standard**. The reference standard is the best available method for establishing the presence or absence of the target condition. An accuracy study can rely on one or more reference standards.

If test results are categorized as either positive or negative, the cross tabulation of the index test results against those of the reference standard can be used to estimate the **sensitivity** of the index test (the proportion of participants *with* the target condition who have a positive index test), and its **specificity** (the proportion *without* the target condition who have a negative index test). From this cross tabulation (sometimes referred to as the contingency or “2x2” table), several other accuracy statistics can be estimated, such as the positive and negative **predictive values** of the test. Confidence intervals around estimates of accuracy can then be calculated to quantify the statistical **precision** of the measurements.

If the index test results can take more than two values, categorization of test results as positive or negative requires a **test positivity cut-off**. When multiple such cut-offs can be defined, authors can report a receiver operating characteristic (ROC) curve which graphically represents the combination of sensitivity and specificity for each possible test positivity cut-off. The **area under the ROC curve** informs in a single numerical value about the overall diagnostic accuracy of the index test.

The **intended use** of a medical test can be diagnosis, screening, staging, monitoring, surveillance, prediction or prognosis. The **clinical role** of a test explains its position relative to existing tests in the clinical pathway. A replacement test, for example, replaces an existing test. A triage test is used before an existing test; an add-on test is used after an existing test.

Besides diagnostic accuracy, several other outcomes and statistics may be relevant in the evaluation of medical tests. Medical tests can also be used to classify patients for purposes other than diagnosis, such as staging or prognosis. The STARD list was not explicitly developed for these other outcomes, statistics, and study types, although most STARD items would still apply.

### DEVELOPMENT

This STARD list was released in 2015. The 30 items were identified by an international expert group of methodologists, researchers, and editors. The guiding principle in the development of STARD was to select items that, when reported, would help readers to judge the potential for bias in the study, to appraise the applicability of the study findings and the validity of conclusions and recommendations. The list represents an update of the first version, which was published in 2003.

More information can be found on [http://www.equator-network.org/reporting-guidelines/stard](http://www.equator-network.org/reporting-guidelines/stard/).
