# Supplementary material for: DNA Microarray Platform for Detection and Surveillance of Viruses Transmitted by Small Mammals and Arthropods
Source: PLoS Negl Trop Dis. 2016 Sep 21;10(9):e0005017. doi: 10.1371/journal.pntd.0005017 (PMC5031435; doi:10.1371/journal.pntd.0005017)
Supplement: S1 Table — All viruses targeted by SMAvirusChip v1 and v2. (PDF) [file pntd.0005017.s002.pdf]

Virus name / RoboArboChip v1	Virus name / RoboArboChip v2	Genus	Family
Abu Hammad virus	Abu Hammad virus	Nairovirus	Bunyaviridae
Abu Mina virus	Abu Mina virus	Nairovirus	Bunyaviridae
Adelaide River virus	Adelaide River virus	Ephemerovirus	Rhabdoviridae
African horsesickness virus	African horsesickness virus	Orbivirus	Reoviridae
African swine fever virus	African swine fever virus	Asfarvirus	Asfarviridae
Aguacate virus	Aguacate virus	Phlebovirus	Bunyaviridae
Aino virus	Aino virus	Orthobunyavirus	Bunyaviridae
Akabane virus	Akabane virus	Orthobunyavirus	Bunyaviridae
Alenquer virus	Alenquer virus	Phlebovirus	Bunyaviridae
Alkhurma hemorrhagic fever virus	Alkhurma hemorrhagic fever virus	Flavivirus	Flaviviridae
Allpahuayo virus	Allpahuayo virus	Arenavirus	Arenaviridae
Altai virus	Altai virus	Hantavirus	Bunyaviridae
Alto Paraguay hantavirus	Alto Paraguay hantavirus	Hantavirus	Bunyaviridae
Amapari virus	Amapari virus	Arenavirus	Arenaviridae
Amur virus	Amur virus	Hantavirus	Bunyaviridae
ANAJ Hantavirus	ANAJ Hantavirus	Hantavirus	Bunyaviridae
Andes virus	Andes virus	Hantavirus	Bunyaviridae
Anhembi virus	Anhembi virus	Orthobunyavirus	Bunyaviridae
Anopheles A virus	Anopheles A virus	Orthobunyavirus	Bunyaviridae
Anopheles B virus	Anopheles B virus	Orthobunyavirus	Bunyaviridae
Apeu virus	Apeu virus	Orthobunyavirus	Bunyaviridae
Apoi virus	Apoi virus	Flavivirus	Flaviviridae
Araraquara virus	Araraquara virus	Hantavirus	Bunyaviridae
Araucaria virus	Araucaria virus	Hantavirus	Bunyaviridae
Arbia virus	Arbia virus	Phlebovirus	Bunyaviridae
Ariquemes virus	Ariquemes virus	Phlebovirus	Bunyaviridae
Armero virus	Armero virus	Flavivirus	Flaviviridae
Aroa virus	Aroa virus	Flavivirus	Flaviviridae
Artybash virus	Artybash virus	Hantavirus	Bunyaviridae
Arumowot virus	Arumowot virus	Phlebovirus	Bunyaviridae
Asama virus	Asama virus	Hantavirus	Bunyaviridae
Ash River virus	Ash River virus	Hantavirus	Bunyaviridae
Aura virus	Aura virus	Alphavirus	Togaviridae
Babanki virus	Babanki virus	Alphavirus	Togaviridae
Bagaza virus	Bagaza virus	Flavivirus	Flaviviridae
Bandia virus	Bandia virus	Nairovirus	Bunyaviridae
Bannavirus	Banna virus	Seadornavirus	Reoviridae
Banzi virus	Banzi virus	Flavivirus	Flaviviridae
Barmah Forest virus	Barmah Forest virus	Alphavirus	Togaviridae
Batai virus	Batai virus	Orthobunyavirus	Bunyaviridae
Batama virus	Batama virus	Orthobunyavirus	Bunyaviridae
Batu Cave virus	Batu Cave virus	Flavivirus	Flaviviridae
Bayou virus	Bayou virus	Hantavirus	Bunyaviridae
Bear Canyon virus	Bear Canyon virus	Arenavirus	Arenaviridae
Bebaru virus	Bebaru virus	Alphavirus	Togaviridae
Belterra virus	Belterra virus	Phlebovirus	Bunyaviridae
Bermejo virus	Bermejo virus	Hantavirus	Bunyaviridae
Berrimah virus	Berrimah virus	Ephemerovirus	Rhabdoviridae
Bhanja virus	Bhanja virus	Phlebovirus	Bunyaviridae
Big brushy tank virus	Big brushy tank virus	Arenavirus	Arenaviridae
Birao virus	Birao virus	Orthobunyavirus	Bunyaviridae
Black Creek Canal virus	Black Creek Canal virus	Hantavirus	Bunyaviridae
Black Mesa virus	Black Mesa virus	Arenavirus	Arenaviridae
Blue River virus	Blue River virus	Hantavirus	Bunyaviridae
Bluetongue virus	Bluetongue virus	Orbivirus	Reoviridae
Boraceia virus	Boraceia virus	Orthobunyavirus	Bunyaviridae
Bouboui virus	Bouboui virus	Flavivirus	Flaviviridae

Bovine ephemeral fever virus	Bovine ephemeral fever virus	Ephemerovirus	Rhabdoviridae
Bozo virus	Bozo virus	Orthobunyavirus	Bunyaviridae
Broadhaven virus	Broadhaven virus	Orbivirus	Reoviridae
Buenaventura virus	Buenaventura virus	Phlebovirus	Bunyaviridae
Buggy Creek virus	Buggy Creek virus	Alphavirus	Togaviridae
Bujaru virus	Bujaru virus	Phlebovirus	Bunyaviridae
Bukalasa bat virus	Bukalasa bat virus	Flavivirus	Flaviviridae
Bunyamwera virus	Bunyamwera virus	Orthobunyavirus	Bunyaviridae
Bussuquara virus	Bussuquara virus	Flavivirus	Flaviviridae
Bwamba virus	Bwamba virus	Orthobunyavirus	Bunyaviridae
Cabassou virus	Cabassou virus	Alphavirus	Togaviridae
Cacao virus	Cacao virus	Phlebovirus	Bunyaviridae
Cache Valley virus	Cache Valley virus	Orthobunyavirus	Bunyaviridae
Cachoeira Porteira virus	Cachoeira Porteira virus	Orthobunyavirus	Bunyaviridae
Cacipacore virus	Cacipacore virus	Flavivirus	Flaviviridae
California encephalitis virus	California encephalitis virus	Orthobunyavirus	Bunyaviridae
Calovo virus	Calovo virus	Orthobunyavirus	Bunyaviridae
Camp Ripley virus	Camp Ripley virus	Hantavirus	Flaviviridae
Candiru virus	Candiru virus	Phlebovirus	Bunyaviridae
Cano - Delgadito virus	Cano - Delgadito virus	Hantavirus	Bunyaviridae
Cao Bang virus	Cao Bang virus	Hantavirus	Bunyaviridae
Carajas virus	Carajas virus	Vesiculovirus	Rhabdoviridae
Caraparu	Caraparu virus	Orthobunyavirus	Bunyaviridae
Carey Island virus	Carey Island virus	Flavivirus	Flaviviridae
Carrizal virus	Carrizal virus	Hantavirus	Bunyaviridae
Castelo dos Sonhos virus	Castelo dos Sonhos virus	Hantavirus	Bunyaviridae
Catacamas virus	Catacamas virus	Hantavirus	Bunyaviridae
Catarina virus	Catarina virus	Arenavirus	Arenaviridae
Catch-me-cave virus	Catch-me-cave virus	Phlebovirus	Bunyaviridae
Cell fusing agent virus	Cell fusing agent virus	Flavivirus	Flaviviridae
Chagres virus	Chagres virus	Phlebovirus	Bunyaviridae
Chandipura virus	Chandipura virus	Vesiculovirus	Rhabdoviridae
Chandiru virus	Chandiru virus	Phlebovirus	Bunyaviridae
Chapare virus	Chapare virus	Arenavirus	Arenaviridae
Chatanga virus	Chatanga virus	Orthobunyavirus	Bunyaviridae
Chikungunya virus	Chikungunya virus	Alphavirus	Togaviridae
Choclo virus	Choclo virus	Hantavirus	Bunyaviridae
Cholul virus	Cholul virus	Orthobunyavirus	Bunyaviridae
Chuzan Virus	Chuzan Virus	Orbivirus	Reoviridae
Cocal virus	Cocal virus	Vesiculovirus	Rhabdoviridae
Colorado tick fever virus	Colorado tick fever virus	Coltivirus	Reoviridae
	Convict Creek 107 virus	Hantavirus	Bunyaviridae
Corfou virus	Corfou virus	Phlebovirus	Bunyaviridae
Cowbone Ridge virus	Cowbone Ridge virus	Flavivirus	Flaviviridae
Crimean-Congo hemorrhagic fever virus	Crimean-Congo hemorrhagic fever virus	Nairovirus	Bunyaviridae
Cupixi virus	Cupixi virus	Arenavirus	Arenaviridae
Cy1014 virus	Cy1014 virus	Flavivirus	Flaviviridae
D' Aguilar virus	D' Aguilar virus	Orbivirus	Reoviridae
Dakar bat virus	Dakar bat virus	Flavivirus	Flaviviridae
Dandenong virus	Dandenong virus	Arenavirus	Arenaviridae
Deer tick virus	Deer tick virus	Flavivirus	Flaviviridae
Dengue virus type 1	Dengue virus type 1	Flavivirus	Flaviviridae
Dengue virus type 2	Dengue virus type 2	Flavivirus	Flaviviridae
Dengue virus type 3	Dengue virus type 3	Flavivirus	Flaviviridae
Dengue virus type 4	Dengue virus type 4	Flavivirus	Flaviviridae
Dobrava-Belgrade virus	Dobrava-Belgrade virus	Hantavirus	Bunyaviridae
Douglas virus	Douglas virus	Orthobunyavirus	Bunyaviridae
Dugbe virus	Dugbe virus	Nairovirus	Bunyaviridae

Durania virus	Durania virus	Phlebovirus	Bunyaviridae
Eastern equine encephalitis virus	Eastern equine encephalitis virus	Alphavirus	Togaviridae
Echarte virus	Echarte virus	Phlebovirus	Bunyaviridae
Edge Hill virus	Edge Hill virus	Flavivirus	Flaviviridae
EgAN 1825-61 virus	EgAN 1825-61 virus	Phlebovirus	Bunyaviridae
El Moro Canyon virus	El Moro Canyon virus	Hantavirus	Bunyaviridae
Entebbe bat virus	Entebbe bat virus	Flavivirus	Flaviviridae
Eothenomys miletus hantavirus	Eothenomys miletus Hantavirus	Hantavirus	Bunyaviridae
Epizootic hemorrhagic disease virus 1	Epizootic hemorrhagic disease virus	Orbivirus	Reoviridae
Equine encephalosis virus	Equine encephalosis virus	Orbivirus	Reoviridae
Erve virus	Erve virus	Nairovirus	Bunyaviridae
Eyach virus	Eyach virus	Coltivirus	Reoviridae
Farallon virus	Farallon virus	Nairovirus	Bunyaviridae
Flexal virus	Flexal virus	Arenavirus	Arenaviridae
Fort Morgan virus	Fort Morgan virus	Alphavirus	Togaviridae
Fort Sherman virus	Fort Sherman virus	Orthobunyavirus	Bunyaviridae
Four Corners Hantavirus	Four Corners Hantavirus	Hantavirus	Bunyaviridae
Frijoles virus	Frijoles virus	Phlebovirus	Bunyaviridae
Gabek Forest virus	Gabek Forest virus	Phlebovirus	Bunyaviridae
Gadgets Gully virus	Gadgets Gully virus	Flavivirus	Flaviviridae
Gbagroube virus	Gbagroube virus	Arenavirus	Arenaviridae
Germiston virus	Germiston virus	Orthobunyavirus	Bunyaviridae
Getah virus	Getah virus	Alphavirus	Togaviridae
Granada virus	Granada virus	Phlebovirus	Bunyaviridae
Great Island virus	Great Island virus	Orbivirus	Reoviridae
Greek goat encephalitis virus	Greek goat encephalitis virus	Flavivirus	Flaviviridae
Guanarito virus	Guanarito virus	Arenavirus	Arenaviridae
Guaroa virus	Guaroa virus	Orthobunyavirus	Bunyaviridae
Gumbo Limbo virus	Gumbo Limbo virus	Orthobunyavirus	Bunyaviridae
Hantaan virus	Hantaan virus	Hantavirus	Bunyaviridae
Hazara virus	Hazara virus	Nairovirus	Bunyaviridae
Heartland virus	Heartland virus	Phlebovirus	Bunyaviridae
Highlands J virus	Highlands J virus	Alphavirus	Togaviridae
HoJo virus	HoJo virus	Hantavirus	Bunyaviridae
Hokkaido virus	Hokkaido virus	Hantavirus	Bunyaviridae
Huitzilac virus	Huitzilac virus	Hantavirus	Bunyaviridae
I612045 virus	I612045 virus	Orthobunyavirus	Bunyaviridae
Iaco virus	Iaco virus	Orthobunyavirus	Bunyaviridae
Icoaraci virus	Icoaraci virus	Phlebovirus	Bunyaviridae
Iguape virus	Iguape virus	Flavivirus	Flaviviridae
Ilesha virus	Ilesha virus	Orthobunyavirus	Bunyaviridae
Ilheus virus	Ilheus virus	Flavivirus	Flaviviridae
Imjin virus	Imjin virus	Hantavirus	Bunyaviridae
Ingwavuma virus	Ingwavuma virus	Orthobunyavirus	Bunyaviridae
Inkoo virus	Inkoo virus	Orthobunyavirus	Bunyaviridae
Ippy virus	Ippy virus	Arenavirus	Arenaviridae
Isfahan virus	Isfahan virus	Vesiculovirus	Rhabdoviridae
Isla Vista virus	Isla Vista virus	Hantavirus	Bunyaviridae
Israel turkey meningoencephalomyelitis	Israel turkey meningoencephalomyelitis	Flavivirus	Flaviviridae
Itaituba virus	Itaituba virus	Phlebovirus	Bunyaviridae
Itaporanga virus	Itaporanga virus	Phlebovirus	Bunyaviridae
Itaqui virus	Itaqui virus	Orthobunyavirus	Bunyaviridae
Ixcanal virus	Ixcanal virus	Phlebovirus	Bunyaviridae
Jabora virus	Jabora virus	Hantavirus	Bunyaviridae
Jacunda Virus	Jacunda Virus	Flavivirus	Flaviviridae
Jamestown Canyon virus	Jamestown Canyan virus	Orthobunyavirus	Bunyaviridae
Japanese encephalitis virus	Japanese encephalitis virus	Flavivirus	Flaviviridae
Jeju virus	Jeju virus	Hantavirus	Bunyaviridae

Jerry Slough virus	Jerry Slough virus	Orthobunyavirus	Bunyaviridae
Joa virus	Joa virus	Phlebovirus	Bunyaviridae
Jugra virus	Jugra virus	Flavivirus	Flaviviridae
Junin virus	Junin virus	Arenavirus	Arenaviridae
Jurona virus	Jurona virus	Vesiculovirus	Rhabdoviridae
Jutiapa virus	Jutiapa virus	Flavivirus	Flaviviridae
Kadam virus	Kadam virus	Flavivirus	Flaviviridae
Kadipiro virus	Kadipiro virus	Seadornavirus	Reoviridae
Kaeng Khoi virus	Kaeng Khoi virus	Orthobunyavirus	Bunyaviridae
Kairi virus	Kairi virus	Orthobunyavirus	Bunyaviridae
Karimabad virus	Karimabad virus	Phlebovirus	Bunyaviridae
Karshi virus	Karshi virus	Flavivirus	Flaviviridae
Kedougou virus	Kedougou virus	Flavivirus	Flaviviridae
Kemerovo virus	Kemerovo virus	Orbivirus	Reoviridae
Kenkeme virus	Kenkeme virus	Hantavirus	Bunyaviridae
Keystone virus	Keystone virus	Orthobunyavirus	Bunyaviridae
Khabarovsk	Khabarovsk	Hantavirus	Bunyaviridae
Kimberley virus	Kimberley virus	Ephemerovirus	Rhabdoviridae
Klamath virus	Klamath virus	Vesiculovirus	Rhabdoviridae
Kodoko virus	Kodoko virus	Arenavirus	Arenaviridae
Kokobera virus	Kokobera virus	Flavivirus	Flaviviridae
Koutango virus	Koutango virus	Flavivirus	Flaviviridae
Kunjin virus	Kunjin virus	Flavivirus	Flaviviridae
Kupe virus	Kupe virus	Nairovirus	Bunyaviridae
La Crosse virus	La Crosse virus	Orthobunyavirus	Bunyaviridae
Laguna Negra virus	Laguna Negra virus	Hantavirus	Bunyaviridae
Langat virus	Langat virus	Flavivirus	Flaviviridae
Lassa virus	Lassa virus	Arenavirus	Arenaviridae
Latino virus	Latino virus	Arenavirus	Arenaviridae
Leanyer virus	Leanyer virus	Orthobunyavirus	Bunyaviridae
Lechiguanas virus	Lechiguanas virus	Hantavirus	Bunyaviridae
Liao ning virus	Liao ning virus	Seadornavirus	Reoviridae
Lipovnik virus	Lipovnik virus	Orbivirus	Reoviridae
Lokern virus	Lokern virus	Orthobunyavirus	Bunyaviridae
Louping ill virus	Louping ill virus	Flavivirus	Flaviviridae
Lujo virus	Lujo virus	Arenavirus	Arenaviridae
Lumbo virus	Lumbo virus	Orthobunyavirus	Bunyaviridae
Luna virus	Luna virus	Arenavirus	Arenaviridae
Lunk virus	Lunk virus	Arenavirus	Arenaviridae
Lymphocytic choriomeningitis virus	Lymphocytic choriomeningitis virus	Arenavirus	Arenaviridae
M'Poko virus	M'Poko virus	Orthobunyavirus	Bunyaviridae
Macaua virus	Macaua virus	Orthobunyavirus	Bunyaviridae
Machupo virus	Machupo virus	Arenavirus	Arenaviridae
Maciel virus	Maciel virus	Hantavirus	Bunyaviridae
Madrid virus	Madrid virus	Orthobunyavirus	Bunyaviridae
Maguari virus	Maguari virus	Orthobunyavirus	Bunyaviridae
Main Drain virus	Main Drain virus	Orthobunyavirus	Bunyaviridae
Malakal virus	Malakal virus	Ephemerovirus	Rhabdoviridae
Maldonado virus	Maldonado virus	Phlebovirus	Bunyaviridae
Malpais Spring virus	Malpais Spring virus	Vesiculovirus	Rhabdoviridae
Maporal virus	Maporal virus	Hantavirus	Bunyaviridae
Maraba virus	Maraba virus	Vesiculovirus	Rhabdoviridae
Maripa hantavirus	Maripa Hantavirus	Hantavirus	Bunyaviridae
Marituba virus	Marituba virus	Orthobunyavirus	Bunyaviridae
Massilia virus	Massilia virus	Phlebovirus	Bunyaviridae
Mayaro virus	Mayaro virus	Alphavirus	Togaviridae
Meaban virus	Meaban virus	Flavivirus	Flaviviridae
Melao virus	Melao virus	Orthobunyavirus	Bunyaviridae

Menekre virus	Menekre virus	Arenavirus	Arenaviridae
Merino Walk virus	Merino Walk virus	Arenavirus	Arenaviridae
Middelburg virus	Middelburg virus	Alphavirus	Togaviridae
Mobala virus	Mobala virus	Arenavirus	Arenaviridae
Modoc virus	Modoc virus	Flavivirus	Flaviviridae
Montana myotis leukoencephalitis virus	Montana myotis leukoencephalitis virus	Flavivirus	Flaviviridae
Montano virus	Montano virus	Hantavirus	Bunyaviridae
Mopia virus	Mopia virus	Arenavirus	Arenaviridae
Morogoro virus	Morogoro virus	Arenavirus	Arenaviridae
Morro Bay virus	Morro Bay virus	Ephemerovirus	Rhabdoviridae
Morumbi virus	Morumbi virus	Phlebovirus	Bunyaviridae
Mount Elgon bat virus	Mount Elgon bat virus	Vesiculovirus	Rhabdoviridae
Mucambo virus	Mucambo virus	Alphavirus	Togaviridae
Mucura virus	Mucura virus	Phlebovirus	Bunyaviridae
Muju virus	Muju virus	Hantavirus	Bunyaviridae
	Muleshoe virus	Hantavirus	Bunyaviridae
Munguba virus	Munguba virus	Phlebovirus	Bunyaviridae
Murray Valley encephalitis virus	Murray Valley encephalitis virus	Flavivirus	Flaviviridae
Murutucu virus	Murutucu virus	Orthobunyavirus	Bunyaviridae
Nairobi sheep disease virus	Nairobi sheep disease virus	Nairovirus	Bunyaviridae
Naranjal virus	Naranjal virus	Flavivirus	Flaviviridae
Ndumu virus	Ndumu virus	Alphavirus	Togaviridae
Neembucu Hantavirus	Neembucu Hantavirus	Hantavirus	Bunyaviridae
	Negishi virus	Flavivirus	Flaviviridae
Nepuyo virus	Nepuyo virus	Orthobunyavirus	Bunyaviridae
New York virus	New York virus	Hantavirus	Bunyaviridae
Newfound Gap hantavirus	Newfound Gap Hantavirus	Hantavirus	Bunyaviridae
Ngari virus	Ngari virus	Orthobunyavirus	Bunyaviridae
Nique virus	Nique virus	Phlebovirus	Bunyaviridae
Nola virus	Nola virus	Orthobunyavirus	Bunyaviridae
North American arenavirus	North American arenavirus	Arenavirus	Arenaviridae
Northway virus	Northway virus	Orthobunyavirus	Bunyaviridae
Nova virus	Nova virus	Hantavirus	Bunyaviridae
Ntaya virus	Ntaya virus	Phlebovirus	Bunyaviridae
Nyabira virus	Nyabira virus	Orbivirus	Reoviridae
Nyando virus	Nyando virus	Orthobunyavirus	Bunyaviridae
Odrenisrou virus	Odrenisrou virus	Phlebovirus	Bunyaviridae
Oliveros virus	Oliveros virus	Arenavirus	Arenaviridae
Omsk hemorrhagic fever virus	Omsk hemorrhagic fever virus	Flavivirus	Flaviviridae
Oran virus	Oran virus	Hantavirus	Bunyaviridae
Oriboca virus	Oriboca virus	Orthobunyavirus	Bunyaviridae
Oriximina virus	Oriximina virus	Phlebovirus	Bunyaviridae
Orogrande virus	Orogrande virus	Arenavirus	Arenaviridae
Oropouche virus	Oropouche virus	Orthobunyavirus	Bunyaviridae
Oxbow virus	Oxbow virus	Phlebovirus	Bunyaviridae
Oyo virus	Oyo virus	Orthobunyavirus	Bunyaviridae
Palma virus	Palma virus	Phlebovirus	Bunyaviridae
Pampa virus	Pampa virus	Arenavirus	Arenaviridae
Parana virus	Parana virus	Arenavirus	Arenaviridae
Pata virus	Pata virus	Orbivirus	Reoviridae
Peaton virus	Peaton virus	Orthobunyavirus	Bunyaviridae
Pergamino virus	Pergamino virus	Hantavirus	Bunyaviridae
Perinet virus	Perinet virus	Vesiculovirus	Rhabdoviridae
Phnom Penh bat virus	Phnom Penh bat virus	Flavivirus	Flaviviridae
Pichinde virus	Pichinde virus	Arenavirus	Arenaviridae
Pike fry rhabdovirus	Pike fry rhabdovirus	Vesiculovirus	Rhabdoviridae
Pirital virus	Pirital virus	Arenavirus	Arenaviridae
Piry virus	Piry virus	Vesiculovirus	Rhabdoviridae

Playa de Oro hantavirus	Playa de Oro Hantavirus	Hantavirus	Bunyaviridae
Playas virus	Playas virus	Orthobunyavirus	Bunyaviridae
Pongola virus	Pongola virus	Orthobunyavirus	Bunyaviridae
Potiskum virus	Potiskum virus	Flavivirus	Flaviviridae
Powassan virus	Powassan virus	Flavivirus	Flaviviridae
Precarious point virus	Precarious point virus	Phlebovirus	Bunyaviridae
Prospect Hill virus	Prospect Hill virus	Hantavirus	Bunyaviridae
Punique virus	Punique virus	Phlebovirus	Bunyaviridae
Punta Salinas virus	Punta Salinas virus	Nairovirus	Bunyaviridae
Punta Toro virus	Punta Toro virus	Phlebovirus	Bunyaviridae
Puumala virus	Puumala virus	Hantavirus	Bunyaviridae
Qalyub virus	Qalyub virus	Nairovirus	Bunyaviridae
Qiandao Lake virus	Qiandao Lake virus	Hantavirus	Bunyaviridae
Raza virus	Raza virus	Nairovirus	Bunyaviridae
Restan virus	Restan virus	Orthobunyavirus	Bunyaviridae
Rift Valley fever virus	Rift Valley fever virus	Phlebovirus	Bunyaviridae
Rio Bravo virus	Rio Bravo virus	Flavivirus	Flaviviridae
Rio Grande virus	Rio Grande virus	Phlebovirus	Bunyaviridae
Rio Mamore virus	Rio Mamore virus	Hantavirus	Bunyaviridae
Rio Negro virus	Rio Negro virus	Alphavirus	Togaviridae
Rio Segundo virus	Rio Segundo virus	Hantavirus	Bunyaviridae
Rocio virus	Rocio virus	Flavivirus	Flaviviridae
Rockport virus	Rockport virus	Hantavirus	Bunyaviridae
Ross River virus	Ross River virus	Alphavirus	Togaviridae
Royal Farm virus	Royal Farm virus	Flavivirus	Flaviviridae
Saaremaa virus	Saaremaa virus	Hantavirus	Bunyaviridae
Sabia virus	Sabia virus	Arenavirus	Arenaviridae
Saboya virus	Saboya virus	Flavivirus	Flaviviridae
Sagiyama virus	Sagiyama virus	Alphavirus	Togaviridae
Saint Croix River virus	Saint Croix River virus	Orbivirus	Reoviridae
Sal Vieja virus	Sal Vieja virus	Flavivirus	Flaviviridae
Salehabad virus	Salehabad virus	Phlebovirus	Bunyaviridae
	Salmon pancreas disease virus	Alphavirus	Togaviridae
Salobo virus	Salobo virus	Phlebovirus	Bunyaviridae
San Angelo virus	San Angelo virus	Orthobunyavirus	Bunyaviridae
Sandfly fever Naples virus	Sandfly fever Naples virus	Phlebovirus	Bunyaviridae
Sandfly fever sicilian virus	Sandfly fever sicilian virus	Phlebovirus	Bunyaviridae
Sandfly Sicilian Turkey virus	Sandfly Sicilian Turkey virus	Phlebovirus	Bunyaviridae
Sangassou virus	Sangassou virus	Hantavirus	Bunyaviridae
Sango virus	Sango virus	Orthobunyavirus	Bunyaviridae
Sapporo rat virus	Sapporo rat virus	Hantavirus	Bunyaviridae
Sathuperi virus	Sathuperi virus	Orthobunyavirus	Bunyaviridae
Saumarez Reef virus	Saumarez Reef virus	Flavivirus	Flaviviridae
Schmallenberg virus	Schmallenberg virus	Orthobunyavirus	Bunyaviridae
Seewis virus	Seewis virus	Hantavirus	Bunyaviridae
Semliki Forest virus	Semliki Forest virus	Alphavirus	Togaviridae
Seoul virus	Seoul virus	Hantavirus	Bunyaviridae
Sepik virus	Sepik virus	Flavivirus	Flaviviridae
Serang virus	Serang virus	Hantavirus	Bunyaviridae
Serra do Navio virus	Serra do Navio virus	Orthobunyavirus	Bunyaviridae
Serra Norte virus	Serra Norte virus	Phlebovirus	Bunyaviridae
Shamonda virus	Shamonda virus	Orthobunyavirus	Bunyaviridae
Shokwe virus	Shokwe virus	Orthobunyavirus	Bunyaviridae
Shuni virus	Shuni virus	Orthobunyavirus	Bunyaviridae
Simbu virus	Simbu virus	Orthobunyavirus	Bunyaviridae
Sin Nombre virus	Sin Nombre virus	Hantavirus	Bunyaviridae
Sindbis virus	Sindbis virus	Alphavirus	Togaviridae
	Sitiawan Virus	Flavivirus	Flaviviridae

Skinner Tank virus	Skinner Tank virus	Arenavirus	Arenaviridae
	Sleeping disease virus	Alphavirus	Togaviridae
Snowshoe hare virus	Snowshoe Hare virus	Orthobunyavirus	Bunyaviridae
Soochong virus	Soochong virus	Hantavirus	Bunyaviridae
Sororoca virus	Sororoca virus	Orthobunyavirus	Bunyaviridae
South River virus	South River virus	Orthobunyavirus	Bunyaviridae
Southern elephant seal virus	Southern elephant seal virus	Alphavirus	Togaviridae
Spondweeni virus	Spondweeni virus	Flavivirus	Flaviviridae
Spring viraemia of carp virus	Spring viraemia of carp virus	Vesiculovirus	Rhabdoviridae
St. Louis encephalitis virus	St. Louis encephalitis virus	Flavivirus	Flaviviridae
Stratford virus	Stratford virus	Flavivirus	Flaviviridae
Tacaiuma virus	Tacaiuma virus	Orthobunyavirus	Bunyaviridae
Tacaribe virus	Tacaribe virus	Arenavirus	Arenaviridae
Tahyna virus	Tahyna virus	Orthobunyavirus	Bunyaviridae
Tamana bat virus	Tamana bat virus	Flavivirus	Flaviviridae
Tamiami virus	Tamiami virus	Arenavirus	Arenaviridae
Tanganya virus	Tanganya virus	Hantavirus	Bunyaviridae
Tehran virus	Tehran virus	Phlebovirus	Bunyaviridae
Tembusu virus	Tembusu virus	Flavivirus	Flaviviridae
Tensaw virus	Tensaw virus	Orthobunyavirus	Bunyaviridae
Tete virus	Tete virus	Orthobunyavirus	Bunyaviridae
Thailand virus	Thailand virus	Hantavirus	Bunyaviridae
Thottapalayam virus	Thottapalayam virus	Hantavirus	Bunyaviridae
Tick-borne encephalitis virus	Tick-borne encephalitis virus	Flavivirus	Flaviviridae
Tillamook virus	Tillamook virus	Nairovirus	Bunyaviridae
Tilligerry virus	Tilligerry virus	Orbivirus	Reoviridae
Tinaroo virus	Tinaroo virus	Orthobunyavirus	Bunyaviridae
Tlacotalpan virus	Tlacotalpan virus	Orthobunyavirus	Bunyaviridae
Tonate virus	Tonate virus	Alphavirus	Togaviridae
Tonto creek virus	Tonto creek virus	Arenavirus	Arenaviridae
Topografov virus	Topografov virus	Hantavirus	Bunyaviridae
Toscana virus	Toscana virus	Phlebovirus	Bunyaviridae
Tribec virus	Tribec virus	Orbivirus	Reoviridae
Trivittatus virus	Trivittatus virus	Orthobunyavirus	Bunyaviridae
Trocara virus	Trocara virus	Alphavirus	Togaviridae
Tucunduba virus	Tucunduba virus	Orthobunyavirus	Bunyaviridae
Tula virus	Tula virus	Hantavirus	Bunyaviridae
Tunari virus	Tunari virus	Hantavirus	Bunyaviridae
Turkish sheep encephalitis virus	Turkish sheep encephalitis virus	Flavivirus	Flaviviridae
Turuna virus	Turuna virus	Phlebovirus	Bunyaviridae
Tyuleniy virus	Tyuleniy virus	Flavivirus	Flaviviridae
Uganda S virus	Uganda S virus	Flavivirus	Flaviviridae
Umatilla virus	Umatilla virus	Orbivirus	Reoviridae
Umbre virus	Umbre virus	Orthobunyavirus	Bunyaviridae
Una virus	Una virus	Alphavirus	Togaviridae
Uriurana virus	Uriurana virus	Phlebovirus	Bunyaviridae
Ussuri virus	Ussuri virus	Hantavirus	Bunyaviridae
Usutu virus	Usutu virus	Flavivirus	Flaviviridae
Utique virus	Utique virus	Phlebovirus	Bunyaviridae
Uukuniemi virus	Uukuniemi virus	Phlebovirus	Bunyaviridae
Venezuelan equine encephalitis virus	Venezuelan equine encephalitis virus	Alphavirus	Togaviridae
Vesicular stomatitis Alagoas virus	Vesicular stomatitis Alagoas virus	Vesiculovirus	Rhabdoviridae
Vesicular stomatitis Indiana virus	Vesicular stomatitis Indiana virus	Vesiculovirus	Rhabdoviridae
Vesicular stomatitis New Jersey virus	Vesicular stomatitis New Jersey virus	Vesiculovirus	Rhabdoviridae
Vinces virus	Vinces virus	Orthobunyavirus	Bunyaviridae
Weldona virus	Weldona virus	Orthobunyavirus	Bunyaviridae
Wesselsbron virus	Wesselsbron virus	Flavivirus	Flaviviridae
West Nile virus	West Nile virus	Flavivirus	Flaviviridae

Western equine encephalitis virus	Western equine encephalitis virus	Alphavirus	Togaviridae
	Whataroa virus	Alphavirus	Togaviridae
Whitewater virus	Whitewater virus	Arenavirus	Arenaviridae
Wyeomyia virus	Wyeomyia virus	Orthobunyavirus	Bunyaviridae
Xingu virus	Xingu virus	Orthobunyavirus	Bunyaviridae
Yaounde virus	Yaounde virus	Flavivirus	Flaviviridae
Yellow fever virus	Yellow fever virus	Flavivirus	Flaviviridae
Yokose virus	Yokose virus	Flavivirus	Flaviviridae
Yug Bogdanovac virus	Yug Bogdanovac virus	Vesiculovirus	Rhabdoviridae
Yunnan Orbivirus	Yunnan Orbivirus	Orbivirus	Reoviridae
Zika virus	Zika virus	Flavivirus	Flaviviridae
